# Etiologic Spectrum and Surgical Epidemiology of Pediatric Cataracts in Kazakhstan: A Three-Year Retrospective Study from Two Tertiary Eye-Care Centers

**DOI:** 10.3390/medicina62071245

**Published:** 2026-06-27

**Authors:** Kairat Ruslanuly, Lukpan Orazbekov, Ardak Auyezova, Neilya Aldasheva, Elmira Kanafyanova, Botagoz Issergepova, Aigerim Tuletova, Gulnaz Kassymbekova, Aibek Kurakbay, Zhansaya Sultanbayeva, Raushan Bakhytbek, Asiya Beysebayeva, Akio Oishi, Dilyara Abessova

**Affiliations:** 1 Science Management Department, Kazakh Eye Research Institute, Almaty 050012, Kazakhstan; aldasheva_n72@mail.ru (N.A.); elmira-kanafyanova@mail.ru (E.K.); bissergepova@mail.ru (B.I.); abessova.dilya98@gmail.com (D.A.); 2 First Ophthalmology Department, Kazakh Eye Research Institute, Almaty 050012, Kazakhstan; lukpan.orazbekov@gmail.com (L.O.); aibekquraqbai@mail.ru (A.K.); zh.sultanbaeva@eyeinst.kz (Z.S.); r.bakytbek@bk.ru (R.B.); 3 Department of Postgraduate Education, Kazakhstan Medical University “Higher School of Public Health”, Almaty 050060, Kazakhstan; mr.ikaay@gmail.com; 4 Pediatrics Inpatient Department, Kazakh Eye Research Institute, Astana 010000, Kazakhstan; a.tuletova@eyeinst.kz (A.T.); gulnazkassymbekova@gmail.com (G.K.); a.beysebayeva@eyeinst.kz (A.B.); 5 Graduate School of Biomedical Sciences, Nagasaki University, Nagasaki 852-8501, Japan; akio.oishi@nagasaki-u.ac.jp

**Keywords:** cataract, cataract/congenital, cataract extraction, eye injuries, ectopia lentis, uveitis

## Abstract

*Background and Objectives*: Pediatric cataract comprises a heterogeneous group of disorders with varying etiologies, laterality patterns, age and gender distributions, and contemporary data from Central Asia remain limited. The study aimed to describe the etiologic spectrum, laterality, age, and gender distribution of pediatric cataract from a two-center tertiary cohort from Kazakhstan. *Materials and Methods*: This retrospective two-center cohort study included all consecutive children aged ≤17 years who underwent cataract surgery between January 2022 and December 2024 at the Astana and Almaty branches of the Kazakh Eye Research Institute. Recorded variables included age at surgery, gender, cataract laterality, and etiology. Etiologies were classified as congenital, traumatic, ectopia lentis-associated, and complicated cataract. No formal hypothesis-testing framework was predefined; analyses were primarily descriptive. *Results*: A total of 528 children (782 eyes) were included. Median age at surgery was 6.5 years (IQR 4.3–9.9 years). Children were distributed as ≤6 years: 294 (55.68%), 7–11 years: 156 (29.55%), and 12–17 years: 78 (14.77%). Operated eyes were distributed as 466 (59.59%), 216 (27.62%), and 100 (12.79%), respectively. Congenital cataract was the predominant etiology, accounting for 412/528 children (78.03%) and 645/782 eyes (82.48%), and was predominantly bilateral (233/412 children, 56.55%). Traumatic cataract accounted for 68/528 children (12.88%) and was uniformly unilateral, with male predominance (53/68, 77.94%). Ectopia lentis-associated cataract accounted for 24/528 children (4.55%) and was more often bilateral (14/24, 58.33%). Complicated cataracts accounted for 24/528 children (4.55%). *Conclusions*: Overall, these findings demonstrate that the etiologic profile of pediatric cataract surgery varies substantially by age, gender, and laterality.

## 1. Introduction

Pediatric cataract is widely recognized as an important cause of childhood blindness and severe visual impairment worldwide, with particularly high lifetime burden because it affects vision development early and requires long-term follow-up and rehabilitation [1]. Epidemiologically, congenital/developmental cataract often drives the caseload in pediatric cataract services, while trauma contributes a preventable, frequently unilateral subset that may become more prominent with increasing age and is often male-skewed in pediatric ocular injury cohorts [1,2]. Cataract in pediatric uveitis is a recognized complication and may require complex surgical decision-making and long-term monitoring [3,4]. Diabetes-related cataract in children and adolescents is comparatively rare in the literature and can present bilaterally, including as an early manifestation in some reports [5,6].

Within Central Asia, published data on pediatric cataract have highlighted delayed presentation for congenital and developmental cases in Kazakhstan and provide a useful regional comparator; however, contemporary etiology surgical data remain limited [7,8]. Therefore, the study aimed to analyze the epidemiological characteristics of pediatric cataracts based on data from two major referral tertiary eye-care centers in Kazakhstan.

## 2. Materials and Methods

### 2.1. Study Design and Settings

This retrospective two-center cohort study adhered to the tenets of the Declaration of Helsinki and was approved by Local Ethics Committee, Expert Council, of Kazakh Eye Research Institute (approval No. 2-2026). Written informed consent for the use of anonymized clinical data was obtained from the parents or legal guardians of all children.

All consecutive pediatric patients aged ≤17 years who underwent cataract surgery between January 2022 and December 2024 at the Astana and Almaty branches of Kazakh Eye Research Institute, two tertiary referral ophthalmic centers in Kazakhstan, were included in the study (Table S1).

### 2.2. Eligibility Criteria

Eligible participants were children aged ≤17 years at the time of surgery with pediatric cataract of any etiology requiring surgical treatment. Exclusions were: (i) eyes already pseudophakic from previous cataract surgery; (ii) eyes with dislocated intraocular lenses; (iii) admissions primarily for posterior capsule opacification removal; and (iv) secondary procedures undertaken for complications related to previous intraocular surgery.

### 2.3. Data Collection and Variables

Data extracted from electronic medical records included: age at surgery, gender, cataract laterality, and cataract etiology. Age at surgery was analyzed as a continuous variable and categorized into three predefined groups: ≤6 years, 7–11 years, and 12–17 years. These strata were selected to compare etiologic patterns across developmental stages. Laterality was defined at the patient level as unilateral or bilateral.

Etiologies were classified into four primary categories based on clinical history and examination findings: (i) congenital cataract (congenital/developmental combined as per clinical classification used in the dataset), (ii) traumatic cataract (post ocular injury), and (iii) complicated cataract (secondary to other ocular/systemic conditions), subclassified as uveitis-related or diabetes-related, ectopia lentis-associated cataract (cataract with non-traumatic lens subluxation due to underlying ocular/systemic disorder).

### 2.4. Statistical Analysis

The primary outcomes were the distribution of cataract etiologies in the overall cohort and across age groups, as well as the laterality pattern associated with each etiology. Continuous data are presented as median and interquartile range (IQR) because age at surgery was not normally distributed (Shapiro–Wilk test). Categorical variables are presented as counts and percentages. To avoid bias related to non-independence of bilateral cases, inferential analyses were performed at the patient level, whereas eye-level data were summarized descriptively.

## 3. Results

### 3.1. Cohort Characteristics

A total of 528 children (782 eyes) met the inclusion criteria. The median age at surgery was 6.5 years (4.3–9.9 years). At the patient level, 294 children (55.68%) were aged ≤6 years, 156 (29.55%) were aged 7–11 years, and 78 (14.77%) were adolescents aged 12–17 years (Table 1). At the eye level, the corresponding distributions were 466 eyes (59.59%), 216 eyes (27.62%), and 100 eyes (12.79%), respectively (Table 2).

Overall, 295 of 528 children (55.87%) were boys and 233 (44.13%) were girls, corresponding to a male-to-female ratio of 1.27:1. At the eye level, 431 of 782 operated eyes (55.12%) were in boys and 351 (44.88%) were in girls, corresponding to a male-to-female ratio of 1.23:1.

Bilateral cataract was present in 254 of 528 children (48.11%), accounting for 508 of 782 eyes (64.96%), whereas unilateral cataract was present in 274 children (51.89%), corresponding to 274 eyes (35.04%).

### 3.2. Etiology and Laterality

Congenital cataract was the predominant etiology in this surgical cohort, accounting for 78.03% of children and 82.48% of eyes. It was more often bilateral than unilateral at the patient level and contributed the majority of bilateral eye involvement in the cohort. The median age at surgery was 5.2 years (3.6–8.2 years).

Traumatic cataract was the second most frequent etiology, accounting for 12.88% of children and 8.70% of eyes. In contrast to congenital cataract, all traumatic cases were unilateral. The median age at surgery was 8.4 years (5.3–11.6 years).

Ectopia lentis-associated cataract and complicated cataract each accounted for 4.55% of children, although ectopia lentis-associated cataract contributed slightly more eyes than complicated cataract (4.86% and 3.96%, respectively). Their laterality patterns differed: ectopia lentis-associated cataract was more often bilateral, whereas complicated cataract was predominantly unilateral at the patient level. The median age at surgery for ectopia lentis-associated and complicated cataracts were 7.0 years (5.0–10.5 years) and 11.0 years (8.0–14.5 years), respectively.

Within the complicated cataract, uveitis-related cataract accounted for 13 children and 15 eyes, and diabetes-related cataract for 11 children and 16 eyes. Uveitis-related cataract was predominantly unilateral, whereas diabetes-related cataract showed a more bilateral pattern at the eye level. The median age at surgery for uveitis-related and diabetes-related cataracts were approximately 10.0 years (7.0–13.5 years) and 13.0 years (10.0–15.5 years), respectively.

### 3.3. Age- and Gender-Specific Distributions

Congenital cataract was concentrated in younger children, with nearly two thirds of affected patients and eyes occurring in the ≤6-year group. By contrast, traumatic cataract peaked in children aged 7–11 years and was rarest in adolescents. Ectopia lentis-associated cataract showed a more even distribution across age groups, although its largest eye-level contribution was also seen in children aged 7–11 years.

Complicated cataracts tended to occur in older children, with three quarters of patients and more than 80% of eyes occurring between 7 and 17 years of age. Within this category, uveitis-related cataract was most frequent in children aged 7–11 years, whereas diabetes-related cataract was concentrated in adolescents, who accounted for more than half of affected patients and nearly two thirds of affected eyes.

Regarding gender distributions, congenital cataract showed a slight male predominance. In contrast, traumatic cataract was strongly male-predominant. Ectopia lentis-associated cataract showed a near-balanced gender distribution, whereas complicated cataracts were more frequent in girls. The female predominance was evident in the uveitis-related subgroup, while diabetes-related cataract showed a milder female predominance.

## 4. Discussion

This study provides a contemporary two-center overview of pediatric cataract surgery in Kazakhstan and shows that the surgical burden remains predominantly driven by congenital cataract, which accounted for more than three quarters of children and more than four fifths of operated eyes in our cohort. At the same time, the cohort was clearly heterogeneous: traumatic cataract represented a smaller but clinically distinct unilateral subgroup, whereas ectopia lentis-associated and complicated cataracts together formed a non-negligible minority of cases. In this respect, our series contributes to the currently limited Central Asian literature and provides a broader characterization of the etiologic spectrum of pediatric cataract requiring surgery.

The predominance of congenital cataract in our cohort is consistent with the general literature showing that congenital/developmental cataract accounts for the majority of pediatric cataract burden worldwide [1,9]. Our findings are also broadly consistent with published data regarding laterality [1,7,9]. In the overall cohort, bilateral and unilateral disease were present in 48.1% and 51.9% of children, respectively, which is close to the broader epidemiologic literature, suggesting that childhood cataract is often relatively balanced between bilateral and unilateral presentation overall. At the same time, when analysis is restricted to congenital cataract, our data show a predominantly bilateral pattern (56.6% of children), which is in line with meta-analytic evidence that congenital cataract tends to be bilateral more often (54.1%) than unilateral [10]. This pattern is also directionally consistent with previous data from Kazakhstan, where congenital/developmental cataract was reported to be predominantly bilateral [7,11].

The age structure of our cohort is likewise comparable with international experience, in that the overall surgical burden was concentrated in younger children, while etiologic composition shifted with age [1,12]. Reviews of pediatric cataract have consistently shown that congenital and infantile cataracts dominate early childhood, whereas acquired causes become relatively more prominent later in childhood and adolescence. Age at surgery in our congenital cataract subgroup was also comparable to previously reported surgical cohorts, although it still indicates that many children underwent surgery beyond infancy. In our study, the median age at surgery for congenital/developmental cataract was 5.2 years. This is close to prior data from the East China series, where the average age at surgery was 4.74 years [13]. An Indian multicenter study likewise reported a mean age at surgery of 74.6 months (about 6.2 years) for congenital/developmental cataract [14]. Although direct comparison is limited by differences in definitions and referral pathways, these numbers collectively suggest that delayed surgery for congenital/developmental cataract remains common in many real-world settings outside highly screened infant populations.

Another meaningful point of comparison concerns gender distribution. In our overall cohort, boys represented 55.9% of children and 55.1% of eyes, indicating a modest male predominance. International studies have not shown a completely uniform sex pattern across all pediatric cataracts; rather, the male-to-female balance appears to depend on the underlying etiologic composition of the cohort. Congenital/developmental cataract series often report either approximate sex balance or a modest male excess, whereas traumatic cataract series consistently show a much stronger predominance in boys [2,9].

Traumatic cataract was the second most frequent etiology in our cohort. In contrast to congenital cataract, all traumatic cataracts in our series were unilateral, with boys comprising 77.9%. The median age at surgery was 8.4 years and the largest proportion of cases was observed in children aged 7–11 years (41.18%), followed by those aged ≤6 years (32.35%). In a tertiary-center study from Turkey, all traumatic cataracts were unilateral, boys accounted for 71.2% of patients, and the most affected age group was 6–10 years [2]. Similarly, an Upper Egypt cohort reported unilateral involvement in all cases, a male proportion of 68%, and the highest frequency in children aged 6–11 years [15]. An eastern China series also found that all traumatic cataracts were unilateral and that boys comprised 82.1% of cases [16]. This male predominance is generally attributed to greater exposure to play-related and outdoor injury risk, especially during school-age years.

Ectopia lentis-associated cataract represented a relatively small but clinically distinct subgroup in our cohort, accounting for 4.6% of children and 4.9% of eyes with median age at surgery of 7.0 years; 58.33% of affected children had bilateral disease, corresponding to 73.68% of eyes. At the patient level, the gender distribution was nearly balanced, with a slight (54.2%) male predominance, and a similar pattern was observed at the eye level (55.3%). These findings are broadly consistent with the international literature in two respects. Published pediatric ectopia lentis cohorts also report a male predominance and bilateral involvement. In a large Indian series of 297 children with ectopia lentis, with a mean age of 8.7 years, boys accounted for 55.9% of patients, and substantially high bilateral involvement was reported in 91.25% of children [17]. Another published Korean pediatric cohort had reported mean ages at surgery of 5.7 years, 85.7% of which received bilateral surgery [18]. Importantly, in our centers, the surgery was not performed solely because ectopia lentis was present, the surgery was indicated for eyes in which the lens was displaced from the visual axis and/or distance and near visual acuity was worse than 0.3 logMAR, or when the condition caused substantial visual discomfort for the child. In this context, our data should be interpreted as a surgical ectopia lentis-associated cataract cohort rather than a full epidemiologic cohort of children with ectopia lentis.

Complicated cataracts accounted for a relatively small but clinically important fraction of our cohort, comprising 4.55% of children and 3.96% of eyes. In contrast to congenital cataract, this subgroup was shifted toward older children, 75.0% of affected patients and 80.6% of eyes were in the 7–17-year age range. It also showed a female predominance, with girls accounting for 62.5% of patients and 64.5% of eyes. Direct comparison with the international literature is more difficult for this category than for congenital, traumatic, or ectopia lentis-associated cataract, because many published pediatric studies do not analyze “complicated cataract” as a single pooled entity. Instead, they usually report cataracts secondary to chronic uveitis, corticosteroid exposure, or systemic metabolic disease separately. Even so, the broad pattern in our data is consistent with published reviews showing that secondary or acquired cataracts due to inflammatory and metabolic causes form a minority of the pediatric cataract burden compared with congenital/developmental cataract, while remaining clinically important because they often arise in medically complex children and may require prolonged multidisciplinary follow-up [1,12].

Uveitis-related cataract accounted for 2.46% of patients and 1.92% of eyes in our cohort, with a clear female and unilateral predominance, 69.23% and 84.62% of patients, respectively, and median age at surgery 10.0 years. The female predominance in our cohort is consistent with the broader pediatric uveitis literature, particularly because juvenile idiopathic arthritis-associated uveitis, one of the major causes of pediatric cataract secondary to inflammation, is more common in girls [19]. Reviews of JIA-associated uveitis report that approximately 75–80% of affected children are female and cataract is recognized as one of the major ocular complications in this group. In a surgical series of pediatric uveitic cataract from Saudi Arabia, the mean age at the surgery was 10.1 years and girls accounted for 65.4% of cases [3]. The main difference between our cohort and Saudi surgical series was predominantly in our series unilateral patients (84.62%), whereas the Saudi surgical series reported bilateral uveitis in 50.0% of patients. The observed difference in laterality should therefore be interpreted cautiously and is likely related to population-specific features and differences in the underlying spectrum of pediatric uveitic disease in our cohort, rather than as evidence against the broader literature. Most pediatric uveitis studies report cataract as part of the broader spectrum of ocular complications and their management, rather than as a separately characterized surgical subgroup [4,20,21]. Therefore, detailed comparative data on children receiving cataract surgery for uveitic cataract remain limited.

Diabetes-related cataract was the least frequent major etiology in our cohort, accounting for 2.08% of children and 2.05% of eyes, with a median age of 13.0 years and a slight female predominance (54.55%). With regard to laterality, diabetes-related cataract was present in both eyes in all affected children, although an indication for bilateral cataract surgery was present in only five of 11 patients (45.45%). Recent reviews and cohort studies have estimated the prevalence of early diabetic cataract in children and adolescents with type 1 diabetes at roughly 0.1–3.4%, while a large German/Austrian registry study reported diabetic cataract in 0.25% of more than 92,000 young people with type 1 diabetes [6,22]. A recent North Indian single-center series found cataract in 1.18% of children with type 1 diabetes [5]. They reported a median age of 9.9 years at cataract diagnosis with 100% of bilateral surgery, an earlier North American surgical series reported a mean age of 11.4 years with 78.6% of bilateral surgery [5,23]. The German/Austrian registry study noted a female preponderance (64.5%) among diabetic cataract [22]. Our data suggests broad agreement with the literature, while also indicating that unilateral presentation can still occur in a tertiary surgical cohort.

A major strength of this study is that it captures the contemporary etiologic spectrum of pediatric cataract surgery across two tertiary referral centers rather than focusing on a single diagnosis. This allowed congenital, traumatic, ectopia lentis-associated, inflammatory, and metabolic cataracts to be examined within the same surgical framework, making the relative contribution of each subgroup more clinically interpretable. A second strength is the explicit separation of patient-level and eye-level analyses. In pediatric cataract, where bilateral disease is common, reporting only one unit of analysis can misrepresent disease burden; our approach therefore improves clinical interpretability and aligns with methodological recommendations in ophthalmic research [24].

Although the Astana and Almaty branches of Kazakh Eye Research Institute are major tertiary ophthalmic surgical referral centers receiving pediatric cataract referrals from multiple regions of Kazakhstan, this two-center cohort should not be interpreted as a population-based nationwide registry. Based on institutional experience, these two centers are likely to capture a substantial proportion of pediatric cataract surgeries performed in the country; however, the exact national coverage cannot be calculated from the available data, because Kazakhstan does not currently have a centralized national pediatric cataract surgical registry, and the number of similar patients treated in regional or private ophthalmic facilities is unknown. Therefore, the present findings should be interpreted as a large tertiary referral surgical profile rather than as a population-based estimate of pediatric cataract incidence in Kazakhstan. Nevertheless, the data remain clinically informative for national-level service planning, particularly for optimizing referral pathways, early detection of congenital cataract, prevention of traumatic cataract, and multidisciplinary management of acquired pediatric cataracts.

The study also has important limitations. First, its retrospective design makes it dependent on the completeness and accuracy of medical records, which limits control over variable definition and introduces the possibility of missing or inconsistently documented clinical details. Second, because this was a tertiary surgical cohort, the findings should not be interpreted as population-based epidemiology or incidence estimates for pediatric cataract in Kazakhstan. Instead, they reflect the case mix that reached two referral centers and proceeded to surgery. This distinction is important, because systematic reviews have shown that childhood cataract epidemiology varies substantially by study design, setting, and case ascertainment [9,25].

## 5. Conclusions

In this two-center pediatric cataract surgical cohort from Kazakhstan, congenital cataract remained the principal indication for pediatric cataract surgery in our two-center cohort and was predominantly bilateral, whereas traumatic cataract represented a distinct unilateral subgroup with strong male predominance. Ectopia lentis-associated and complicated cataracts were less frequent but showed characteristic age-, sex-, and laterality-specific patterns. Overall, these findings demonstrate that the etiologic profile of pediatric cataract surgery varies substantially by age, gender, and laterality, highlighting the need for differentiated clinical pathways that combine early detection of congenital cataract with targeted prevention and multidisciplinary management of acquired forms.

## Figures and Tables

**Table 1:** Distribution of pediatric cataract etiologies by laterality, age group, and gender at the patient level.

Etiology	Patients, *n* (%)	Male Patients, *n* (%)	Female Patients, *n* (%)	Bilateral Patients, *n* (%)	Unilateral Patients, *n* (%)	Patients by Age Group (≤6/7–11/12–17 Years), *n* (%)
Congenital cataract	412 (78.0)	220 (53.4)	192 (46.6)	233 (56.6)	179 (43.4)	255 (61.9)/109 (26.5)/48 (11.7)
Traumatic cataract	68 (12.9)	53 (77.9)	15 (22.1)	0 (0.0)	68 (100.0)	22 (32.4)/28 (41.2)/18 (26.5)
Ectopia lentis-associated cataract	24 (4.5)	13 (54.2)	11 (45.8)	14 (58.3)	10 (41.7)	11 (45.8)/9 (37.5)/4 (16.7)
Complicated cataract †	24 (4.5)	9 (37.5)	15 (62.5)	7 (29.2)	17 (70.8)	6 (25.0)/10 (41.7)/8 (33.3)
—Uveitis-related cataract ‡	13 (2.5)	4 (30.8)	9 (69.2)	2 (15.4)	11 (84.6)	4 (30.8)/7 (53.8)/2 (15.4)
—Diabetes-related cataract ‡	11 (2.1)	5 (45.5)	6 (54.5)	5 (45.5)	6 (54.5)	2 (18.2)/3 (27.3)/6 (54.5)
Total	528 (100.0)	295 (55.9)	233 (44.1)	254 (48.1)	274 (51.9)	294 (55.7)/156 (29.5)/78 (14.8)

Data are presented as *n* (%). Percentages in the “Patients” column are calculated relative to all 528 children. Percentages in the sex, bilateral/unilateral, and age-group columns are calculated within each etiology row. † Complicated cataract included uveitis-related and diabetes-related cataracts. ‡ Uveitis-related and diabetes-related cataracts are shown as descriptive subcategories of complicated cataract and should not be summed again with the parent row in overall totals.

**Table 2:** Distribution of pediatric cataract etiologies by laterality, age group, and gender at the eye level.

Etiology	Eyes, *n* (%)	Male Eyes, *n* (%)	Female Eyes, *n* (%)	Bilateral Eyes, *n* (%)	Unilateral Eyes, *n* (%)	Eyes by Age Group (≤6/7–11/12–17 Years), *n* (%)
Congenital cataract	645 (82.5)	346 (53.6)	299 (46.4)	466 (72.2)	179 (27.8)	425 (65.9)/159 (24.7)/61 (9.5)
Traumatic cataract	68 (8.7)	53 (77.9)	15 (22.1)	0 (0.0)	68 (100.0)	22 (32.4)/28 (41.2)/18 (26.5)
Ectopia lentis-associated cataract	38 (4.9)	21 (55.3)	17 (44.7)	28 (73.7)	10 (26.3)	13 (34.2)/17 (44.7)/8 (21.1)
Complicated cataract †	31 (4.0)	11 (35.5)	20 (64.5)	14 (45.2)	17 (54.8)	6 (19.4)/12 (38.7)/13 (41.9)
—Uveitis-related cataract ‡	15 (1.9)	4 (26.7)	11 (73.3)	4 (26.7)	11 (73.3)	4 (26.7)/8 (53.3)/3 (20.0)
—Diabetes-related cataract ‡	16 (2.0)	7 (43.8)	9 (56.2)	10 (62.5)	6 (37.5)	2 (12.5)/4 (25.0)/10 (62.5)
Total	782 (100.0)	431 (55.1)	351 (44.9)	508 (65.0)	274 (35.0)	466 (59.6)/216 (27.6)/100 (12.8)

Data are presented as *n* (%). Percentages in the “Eyes” column are calculated relative to all 782 eyes. Percentages in the sex, bilateral/unilateral, and age-group columns are calculated within each etiology row. † Complicated cataract included uveitis-related and diabetes-related cataracts. ‡ Uveitis-related and diabetes-related cataracts are shown as descriptive subcategories of complicated cataract and should not be summed again with the parent row in overall totals.

## Data Availability

The datasets of the current study are available from Kairat Ruslanuly upon reasonable request.

## Supplementary Materials

The following supporting information can be downloaded at: https://www.mdpi.com/article/10.3390/medicina62071245/s1, Table S1: Center-wise patient-level distribution of pediatric cataract etiologies in the Astana and Almaty branches.

## Abbreviations

IQR	Interquartile range
JIA	Juvenile idiopathic arthritis
logMAR	Logarithm of the minimum angle of resolution
